# Supplementary material for: Development of a Miniaturized, Automated, and Cost-Effective Device for Enzyme-Linked Immunosorbent Assay
Source: Sensors (Basel). 2025 Aug 24;25(17):5262. doi: 10.3390/s25175262 (PMC12431479; doi:10.3390/s25175262)
Supplement: Supplementary file 1 [file sensors-25-05262-s001.zip › sensors-3795078-supplementary.pdf]

Supplementary Information for

Development of a Miniaturized, Automated, and Cost-Effective Device for Enzyme-Linked Immunosorbent Assay

Majid Aalizadeh [1,2,3,4], Shuo Yang [1,3,4], Suchithra Guntur [5], Vaishnavi Potluri [5], Girish Kulkarni [5] and Xudong Fan [1,3,4,*]

[1] Department of Biomedical Engineering, University of Michigan, Ann Arbor, MI 48109, USA; maalizad@umich.edu (M.A.); yashuo@umich.edu (S.Y.)
[2] Department of Electrical Engineering and Computer Science, University of Michigan, Ann Arbor, MI 48109, USA
[3] Center for Wireless Integrated MicroSensing and Systems (WIMS[2]), University of Michigan, Ann Arbor, MI 48109, USA
[4] Max Harry Weil Institute for Critical Care Research and Innovation, University of Michigan, Ann Arbor, MI 48109, USA
[5] Arborsense Inc., 674 S Wagner Rd., Ann Arbor, MI 48103, USA; sguntur@arborsenseinc.com (S.G.); vpotluri@arborsenseinc.com (V.P.); girishkulkarni@arborsenseinc.com (G.K.)
* Correspondence: xsfan@umich.edu

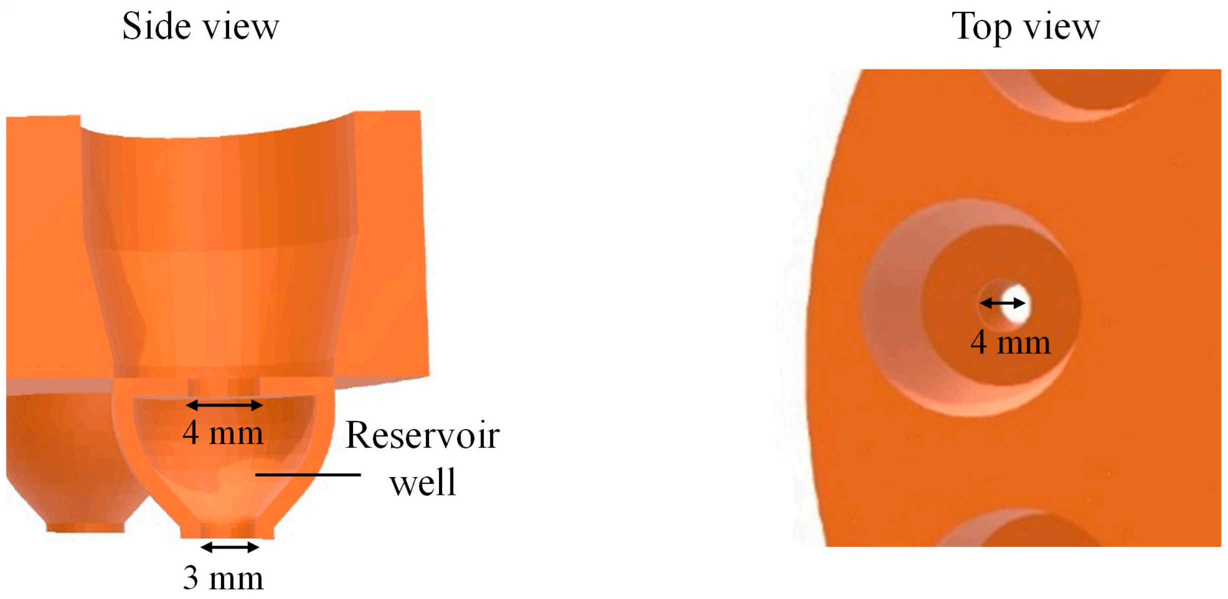

Figure S1. Details of a reservoir well.

Table S1. Costs of the reagents (assume 50 mL consumption, unless otherwise mentioned) and other disposable parts.

Reagent	Concentration/volume used in protocol	Container quantity (conc.)	Vial/bottle cost ($)	Reagent cost per test ($)
Capture antibody	10 mg/mL (in 1X PBS)	0.5 mL (240 mg/mL)	100[1]	0.416
Detection antibody	100 ng/mL (in 3% BSA)	1 mL (3.00 mg/mL)	100[1]	0.166
BSA	3% in 1X PBS (~100 mL used)	600 mL (10% in 1X PBS)	480	0.024
Superblock	No dilution	1 L	207	0.01
Poly-HRP	(0.05 mL used)	0.5 mL	267	0.027
Poly-HRP dilution buffer	No dilution	100 mL	130	0.065
Substrate	No dilution	250 mL	631	0.126
Wash buffer	1X diluted (~1 mL used)	500 mL (25X dilution)	260	0.021
**Total reagents**	-	-	-	**0.855**
1: Estimation based on the retail price of $790 from R&D Systems.				
Reservoir disk				6.7
Microfluidic connector				0.79
Capillary				1
